# Nutrient cross-feeding in the microbial world

**DOI:** 10.3389/fmicb.2014.00350

**Published:** 2014-07-08

**Authors:** Erica C. Seth, Michiko E. Taga

**Affiliations:** Department of Plant and Microbial Biology, University of California, Berkeley, Berkeley, CAUSA

**Keywords:** nutrient cross-feeding, cofactor, microbial interactions, corrinoid, microbial communities

## Abstract

The stability and function of a microbial community depends on nutritional interactions among community members such as the cross-feeding of essential small molecules synthesized by a subset of the population. In this review, we describe examples of microbe–microbe and microbe–host cofactor cross-feeding, a type of interaction that influences the forms of metabolism carried out within a community. Cofactor cross-feeding can contribute to both the health and nutrition of a host organism, the virulence and persistence of pathogens, and the composition and function of environmental communities. By examining the impact of shared cofactors on microbes from pure culture to natural communities, we stand to gain a better understanding of the interactions that link microbes together, which may ultimately be a key to developing strategies for manipulating microbial communities with human health, agricultural, and environmental implications.

## INTRODUCTION

**FIGURE 1:**
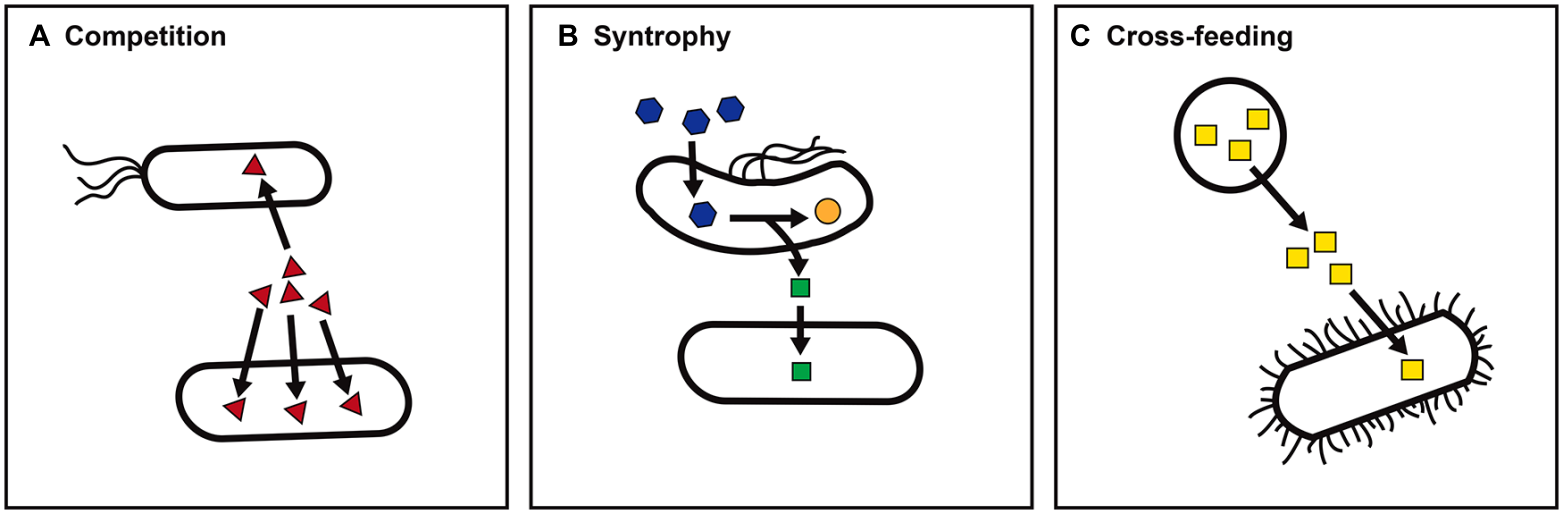
**Nutritional interactions between microbes.** Nutritional interactions may shape a microbe’s metabolic capacity within a microbial community. Three general categories of nutritional interactions are illustrated. **(A)** Nutrient competition. The ability of a microbe to compete for limiting nutrients such as iron (red triangles) may determine survival and persistence within a particular niche. **(B)** Syntrophic metabolism. The consumption of an intermediate or end product such as hydrogen (green squares) by a partner organism allows an otherwise energetically unfavorable reaction, for example, propionate (blue hexagons) to acetate (orange circles) to support growth. **(C)** Nutrient cross-feeding. The presence of a microbe that produces an essential nutrient such as folate (yellow squares) enables auxotrophs to survive.

Life in the microbial world exists as a dynamic network of interactions among microbes that fuels a complex web of interconnected metabolisms (Faust and Raes, 2012). Ignorance of what microbes gain via these interactions impedes our ability to cultivate the vast majority of microbes (Leadbetter, 2003; Tyson and Banfield, 2005). In addition, by failing to elicit a microbe’s full range of metabolic responses to the presence of other organisms, the metabolic potential of microbes grown in isolation may not accurately reflect a microbe’s ecological role (Moller et al., 1998; Traxler et al., 2013). Three broad categories of nutritional interactions that govern a microbe’s ability to carry out specific forms of metabolism within a microbial community are illustrated in **Figure 1**.

The ability to compete for nutrients can determine whether a microbe will be able to persist in a particular niche (Hibbing et al., 2010; Johnson et al., 2012; **Figure 1A**). Specialized ability to gain access to a limiting nutrient (for example, iron acquisition via siderophore production) may give a microbe a competitive advantage in colonization. In contrast, metabolic cooperation between microbes engaged in syntrophic partnerships can allow access to substrates that neither microbe could metabolize alone (**Figure 1B**). For example, the presence of a partner that actively consumes intermediates such as hydrogen (H_2_) allows secondary fermentation of products such as propionate to become energetically favorable (Sieber et al., 2012; Morris et al., 2013). Finally, nutrient cross-feeding – the production of a molecule such as a vitamin or amino acid that is used by both the producing organism and other microbes in the environment – relaxes the metabolic burden on any one microbe in the community (Freilich et al., 2011; **Figure 1C**). Given the complexity of microbial communities, each form of nutritional interaction may have ripple effects on other community members.

## COFACTOR CROSS-FEEDING

In this review, we focus on cross-feeding of cofactors as a model for nutritional interactions between microbes. While cofactor cross-feeding is not necessarily mutualistic, the availability of exogenously produced cofactors can have a profound impact on a microbe’s mode of growth and metabolite production. (Graber and Breznak, 2005; Pedersen et al., 2012). Examining cofactor cross-feeding can inform our understanding of the scope and relevance of metabolic interdependences in microbial communities.

## CROSS-FEEDING OF HEME AND QUINONES ALLOWS LACTIC ACID BACTERIA TO RESPIRE

A striking example of the impact of cofactor cross-feeding on metabolism comes from studies of the lactic acid bacteria, (LAB). Though LAB were long considered to be exclusively dependent on fermentative growth, physiological changes in cultures grown with heme led to the hypothesis that some LAB are capable of aerobic respiration (Bryan-Jones and Whittenbury, 1969). Examination of sequenced environmental and host-associated LAB predicts that the majority have the genetic capacity for respiratory growth if heme, and in some cases, quinones, are supplied. The ability to respire in the presence of these cofactors are supplied has been experimentally verified in several LAB. Respiratory metabolism in LAB has been associated with increased growth rate, long-term survival, and a variety of metabolic changes that may impact other organisms in the environment (Pedersen et al., 2012). For example, in *Lactococcus lactis* (strains of which are frequently isolated from plants) and a number of other LAB, switching from fermentation to respiratory metabolism leads to a decrease in the amount of lactic acid produced and a large increase in the production of acetoin, a volatile compound known to stimulate growth and induce the stringent response in plants (Duwat et al., 2001; Ryu et al., 2003; Rudrappa et al., 2010; Siezen et al., 2010; Jaaskelainen et al., 2013). In the opportunistic pathogen *Streptococcus agalactiae*, the ability to perform respiratory metabolism in the presence of heme and quinones enhances virulence and persistence (Rezaiki et al., 2008). Examples such as these, in which respiratory metabolism is determined by the availability of exogenous cofactors, challenge our understanding of the metabolic capacity of microbes in their natural environments.

## COFACTOR CROSS-FEEDING IN THE TERMITE GUT: *Treponema primitia* AS BOTH A DONOR AND RECIPIENT OF COFACTORS

Investigations of the termite gut-associated bacterium *Treponema primitia* illustrate the complex web of interactions that can exist within a host’s microbiota. *T. primitia* contributes to the host’s nutrition by producing acetate, the major carbon and energy source for the termite, by consuming H_2_ and CO_2_, which are generated as waste products by cellulolytic protists. The process of homoacetogenesis requires folate, yet *T. primitia* is incapable of synthesizing this cofactor (Graber and Breznak, 2004). Given that insects are not known to synthesize folate, and the folate content of the termite’s diet is very low, *T. primitia’*s folate requirements must be fulfilled by other members of the termite gut microbiota. *L. lactis* and *Serratia grimesii* isolated from the termite gut were identified as candidates for this role, as both bacteria secrete 5-formyltetrahydrofolate, the dominant folate compound found in the gut, at levels capable of supporting *T. primitia* growth *in vitro* (Graber and Breznak, 2005).

*T. primitia* is likely to function as a donor of cofactors as well as a recipient. Cofactors produced by *T. primitia* enhance the growth of another important member of the termite gut microbiota, *Treponema azotonutricium*, which supplements the host’s nitrogen-poor wood diet through nitrogen fixation (Graber et al., 2004). In co-culture, *T. primitia* and *T. azotonutricium* achieve higher growth rates and yields than either species grown in isolation. RNA-seq data suggest that in addition to interspecies hydrogen transfer between the two organisms, growth enhancement seen in *T. azotonutricium* may also be influenced by the production of the cofactors biotin, pyridoxal phosphate and co-enzyme A by *T. primitia* (Rosenthal et al., 2011). These findings undoubtedly represent only a small fraction of the complex interactions between members of a very diverse microbiota and provide clues about how microbes that are essential to a host’s nutritional status support and are supported by other members of the microbial community.

## MODELS OF COFACTOR CROSS-FEEDING: CORRINOID SHARING IN MUTUALISTIC PAIRS AND MICROBIAL COMMUNITIES

Of the many documented examples of nutrient cross-feeding, one group of cofactors, the corrinoids, has been particularly informative for understanding cross-feeding mechanisms. Corrinoid-dependent reactions function in diverse metabolic processes across all three domains of life, yet corrinoids are produced solely by a subset of prokaryotes. While the majority of bacteria (75%) are predicted to encode corrinoid-dependent enzymes, at least half of these lack the ability to produce corrinoids *de novo* (Rodionov et al., 2003; Zhang et al., 2009). As such, corrinoid cross-feeding is prevalent and may reflect the advantage of acquiring these complex cofactors from the environment rather than by *de novo* biosynthesis which requires approximately 30 enzymatic steps (Roth et al., 1996; Warren et al., 2002; Zhang et al., 2009). The availability of corrinoids and corrinoid precursors can have profound effects on a microbe’s metabolism and its ability to occupy a specific niche (Matthews, 2009). For instance, the ability to utilize ethanolamine as a sole carbon and nitrogen source relies on the corrinoid-dependent enzyme ethanolamine ammonia lyase, which converts ethanolamine into ammonia and acetaldehyde (Garsin, 2010). In enterohemorrhagic *Escherichia coli,* ethanolamine utilization – enabled by corrinoid cross-feeding – provides a competitive advantage for colonization and persistence in the bovine intestine, a main reservoir for this pathogen (Bertin et al., 2011; Kendall et al., 2012). Ethanolamine utilization is also important in other human pathogens that rely on exogenously produced corrinoids or corrinoid precursors including *Listeria monocytogenes* and *Enterococcus faecalis* (Joseph et al., 2006; Del Papa and Perego, 2008; Garsin, 2010). The mechanism by which corrinoids are released from corrinoid-producing microbes into the environment is unclear. As yet, no active means of corrinoid export has been identified.

**FIGURE 2:**
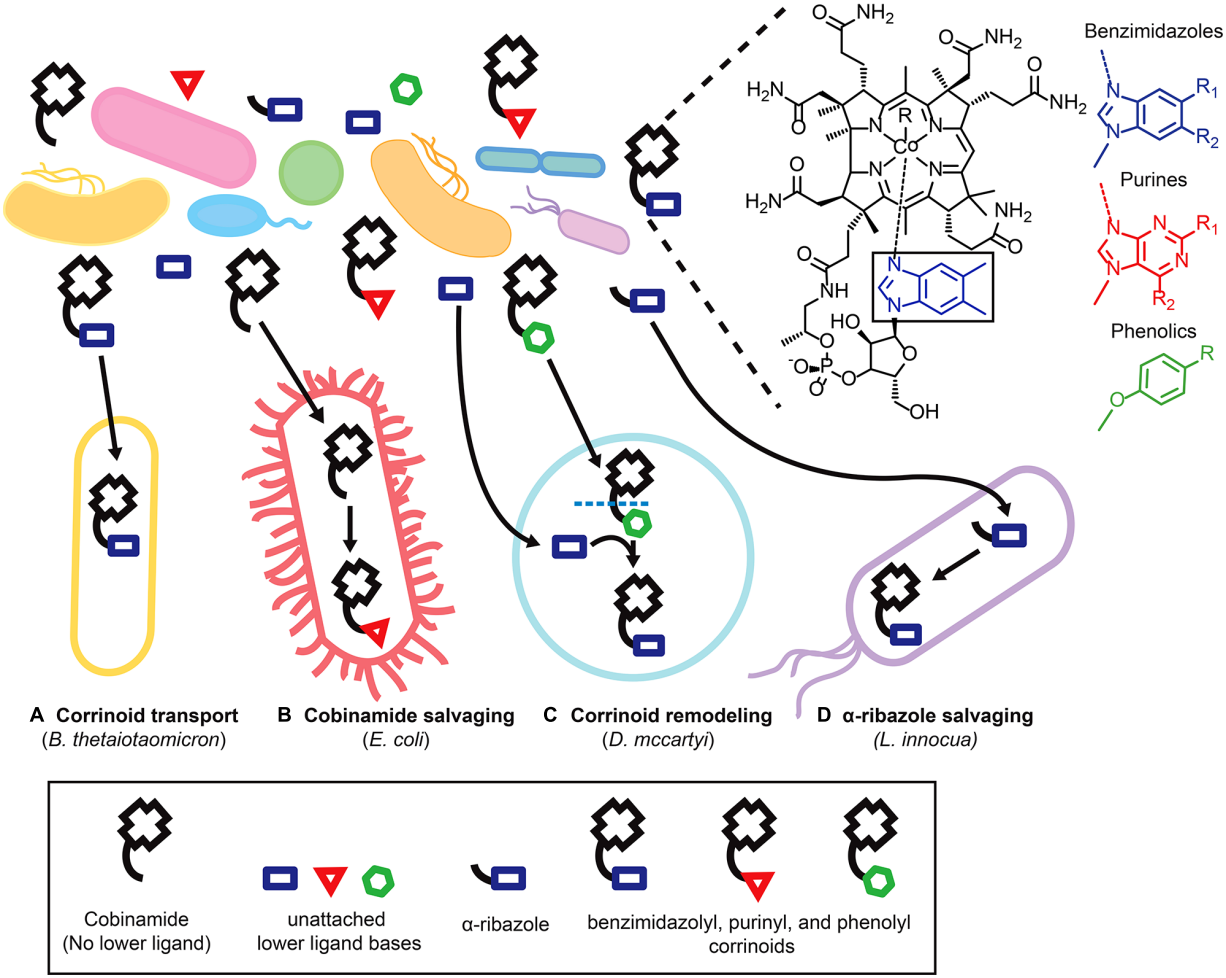
**Microbial strategies for fulfilling corrinoid requirements.** Microbes employ various strategies to obtain specific corrinoids from environments that contain a variety of corrinoids and corrinoid precursors. The structure of cobalamin, a corrinoid with the lower ligand 5,6- dimethylbenzimidazole (boxed) is shown, as are the structures of the three classes of lower ligand bases of other corrinoids. Specific strategies for obtaining corrinoids are illustrated with representative bacteria listed in parentheses. **(A)** Corrinoid transport in *Bacteroides thetaiotaomicron*. **(B)** Cobinamide salvaging in *Escherichia coli*. **(C)** Corrinoid remodeling in *Dehalococcoides mccartyi*. **(D)** α-ribazole salvaging in *Listeria innocua*.

Sixteen distinct corrinoids with structural differences in the lower ligand have been identified and the array of corrinoids that can serve as cofactors differs from organism to organism (Barker et al., 1960; Renz, 1999; Hibbing et al., 2010; Yi et al., 2012). Given that microbial communities have been found to contain multiple corrinoids (Allen and Stabler, 2008; Girard et al., 2009; Men et al., 2014), how do corrinoid-dependent microbes acquire the specific corrinoids that function in their metabolism? As described below, some organisms form mutualisms with corrinoid-producing partners. Others may selectively import corrinoids from the environment or modify imported corrinoids or corrinoid precursors intracellularly (**Figure 2**).

## EXAMPLES OF SPECIFIC MUTUALISTIC INTERACTIONS INVOLVING CORRINOIDS

Phytoplankton are of great ecological importance in their roles in the global carbon cycle, as primary producers in the marine food web, and occasionally as producers of toxins in harmful algal blooms (HABs). Half of eukaryotic algal species are predicted to require exogenously produced corrinoids for growth (Croft et al., 2005; Helliwell et al., 2011). Algal corrinoid requirements can be fulfilled via symbiotic relationships with corrinoid-producing bacteria that colonize the algal surface (Croft et al., 2005; Grant et al., 2014). The availability of corrinoids may play an important role in the occurrence of HABs, as corrinoid auxotrophy is especially prevalent among HAB species (Tang et al., 2010).

Symbiotic relationships can also be fueled by cross-feeding of products of corrinoid-dependent enzymes. Genomic studies of the cicada endosymbiotic bacteria, *Hodgkinia cicadicola* and *Sulcia muelleri*, indicate that the ability to produce a corrinoid is vital for maintaining this tripartite symbiosis (McCutcheon et al., 2009). *H. cicadicola* and *S. muelleri* together produce essential amino acids that are cross-fed between the co-symbionts and provided to the host. The burden of methionine production rests on *H. cicadicola*, which devotes 7% of its 144 kb genome to corrinoid biosynthesis because the methionine synthase it encodes requires a corrinoid cofactor. Interestingly, the corrinoid biosynthesis pathway in *H. cicadicola* is incomplete, and lacks genes involved in the production of precursors common to the biosynthesis of corrinoids as well as other tetrapyrroles such as heme (McCutcheon et al., 2009). We speculate that *H. cicadicola* may acquire tetrapyrrole precursors from the host, completing a loop between organisms which allows maintenance of the symbiotic relationship.

## STRATEGIES USED BY BACTERIA THAT SCAVENGE CORRINOIDS AND THEIR PRECURSORS FROM ENVIRONMENTAL SOURCES

The ability of bacteria to selectively transport specific corrinoids has been largely unexplored, yet may play an important role in ensuring that bacteria acquire the specific corrinoids that function in their metabolism. The corrinoid transporter – BtuBFCD in gram negative bacteria, and BtuFCD in gram positive bacteria - allows bacteria to import corrinoids from the environment (**Figure 2A**) and is present in an estimated 76% of bacterial genomes (Zhang et al., 2009). A recent study of the human gut microbiome found that many bacteria encode multiple copies of the BtuBFCD corrinoid transporter, and that the three homologs of the outer membrane transporter BtuB encoded by the model gut bacterium *Bacteroides thetaiotaomicron*, though apparently redundant, have distinct preferences for different corrinoids (Degnan et al., 2014). The presence of multiple corrinoid transport systems with different affinities for specific corrinoids may allow bacteria to fine-tune their responses to the array of corrinoids present in the environment.

In contrast to obtaining specific corrinoids through selective transport, the ability to remodel corrinoids (that is, to remove the lower ligand of an imported corrinoid and attach a preferred lower ligand in its place) can enable microbes to make use of many different corrinoids present in a community (Gray and Escalante-Semerena, 2009). For instance, our recent work showed that *Dehalococcoides mccartyi,* which has an obligate requirement for exogenously produced corrinoids as cofactors in the reductive dehalogenation of the common groundwater pollutants tetrachloroethene (PCE) and trichloroethene (TCE), is restricted to the use of just three benzimidazolyl corrinoids (Maymo-Gatell et al., 1997; He et al., 2007; Yi et al., 2012). However, if an appropriate benzimidazole lower ligand base is supplied, *D. mccartyi* can fulfill its corrinoid requirements by remodeling other corrinoids (**Figure 2C**; Yi et al., 2012). Since benzimidazolyl corrinoids represent only a fraction of the corrinoids present in a community containing *D. mccartyi,* the ability to remodel corrinoids may be essential to its survival in the environment (Men et al., 2014). Corrinoid remodeling is also performed by some bacteria capable of producing a corrinoid *de novo* (Gray and Escalante-Semerena, 2009), and has been observed in the human gut, where examination of fecal corrinoid profiles before and after ingestion of cobalamin suggests that at least some members of the gut microbiota are engaged in active modification of exogenous corrinoids (Allen and Stabler, 2008).

In addition to importing complete corrinoids (i.e., those containing a lower ligand), some microbes are capable of importing corrinoid precursors from the environment and carrying out the remaining biosynthetic steps to produce a complete corrinoid. For example, though *E. coli* is incapable of *de novo* corrinoid biosynthesis, it possesses the ability to take up cobinamide (a corrinoid precursor that lacks a lower ligand) and convert it to a complete corrinoid through a process known as cobinamide salvaging (**Figure 2B**; Di Girolamo and Bradbeer, 1971; Raux et al., 1996; Escalante-Semerena, 2007). In contrast, *Listeria spp.* encode a nearly complete corrinoid biosynthesis pathway, but apparently lack the genes necessary for lower ligand activation, a step that must be completed before the lower ligand can be attached. *Listeria innocua* was shown instead to rely on the *cblT* encoded transporter to take up activated lower ligands such as α-ribazole from the environment, which it then phosphorylates and attaches to produce a complete corrinoid (Gray and Escalante-Semerena, 2010; **Figure 2D**). CblT homologs have been identified in a variety of human pathogens including *L. monocytogenes* and *Clostridium botulinum* (Gray and Escalante-Semerena, 2010).

Microbes that produce corrinoids *de novo* can also be affected by the presence of corrinoid precursors. Guided biosynthesis, the process of controlling which corrinoid a microbe produces by providing an excess of a particular lower ligand base, can be a useful tool for determining the specific corrinoids required in different metabolic pathways (Stupperich et al., 1987; Keller et al., 2013). For example, in *Sporomusa ovata*, which produces phenolyl corrinoids, the addition of benzimidazoles and their subsequent incorporation into benzimidazolyl corrinoids inhibits growth to different degrees on substrates that require a corrinoid-dependent methyltransferase for utilization (Stupperich and Konle, 1993; Mok and Taga, 2013). We have detected free lower ligand bases in a variety of environmental samples, which raises the question of whether the production of a free lower ligand base by one organism is capable of affecting the corrinoid production of another; that is, whether guided biosynthesis occurs in nature. Evidence from our recent study of the corrinoid and lower ligand profiles in a TCE-dechlorinating community containing *D. mccartyi* suggests that this indeed may be the case (Men et al., 2014).

## CORRINOIDS AS LYNCHPINS OF MICROBIAL COMMUNITY DYNAMICS?

Given that the majority of bacteria depend on corrinoids for their metabolism, and that only a fraction of available corrinoids may be suitable for use by a particular organism, could it be possible to manipulate microbial communities by targeting corrinoid-dependent metabolism? Recent work has shown that manipulation of the composition of a TCE-dechlorinating community can result in a shift in corrinoid composition, and conversely, that the addition of a corrinoid causes a dramatic shift in marine algal community composition (Koch et al., 2011; Men et al., 2014). With growing interest in developing methods for targeted manipulation of microbial communities to benefit human health and the environment, the utility of altering the composition and/or metabolism occurring in microbial communities via corrinoid supplementation or guided biosynthesis deserves further investigation.

## CONCLUSION

From obligate requirements for exogenously supplied cofactors in *T. primitia* and *D. mccartyi* to unlocking cryptic modes of growth in LAB, examples of cofactor cross-feeding provide a glimpse into the impact of nutritional interactions on individual species as well as entire communities. Cofactor cross-feeding undoubtedly deserves further examination; one need look no further than the vitamin amendments required for microbes growing in pure culture to obtain examples of nutrients that must be supplied by other organisms in their natural environment. Advances in imaging mass spectrometry and multiple “omics” approaches, in combination with pure culture studies of individual microbes and defined consortia, bring us closer to understanding the molecular details of metabolic interactions at new levels (Nakanishi et al., 2011; Phelan et al., 2012).

.

## Conflict of Interest Statement

The authors declare that the research was conducted in the absence of any commercial or financial relationships that could be construed as a potential conflict of interest.
